# 7β-Hydroxy­artemisinin

**DOI:** 10.1107/S1600536808000251

**Published:** 2008-01-09

**Authors:** Paulo B. Carvalho, Bo Liu, Yunshan Wu, John S. Williamson, Mitchell A. Avery

**Affiliations:** a Department of Medicinal Chemistry, University of Mississippi, 417 Faser Hall, University, MS 38677, USA; b National Center for Natural Products Research, Research Institute of Pharmaceutical Sciences, School of Pharmacy, University of Mississippi, University, MS 38677, USA; c Department of Chemistry and Biochemistry, University of Mississippi, University, MS 38677, USA

## Abstract

Crystals of the title compound [systematic name: (3*R*,6*R*,7*S*,8a*R*,9*R*,12a*R*)-7-hydr­oxy-3,6,9-trimethyl­octa­hydro-3,12-ep­oxy[1,2]dioxepino[4,3-*i*]isochromen-10(3*H*)-one], C_15_H_22_O_6_, were obtained from microbial transformation of artemisinin by a culture of *Cunninghamella elegans*. The stereochemistry of the compound is consistent with the spectroscopic findings in previously published works. A weak O—H⋯O hydrogen bond occurs in the crystal structure, together with intermolecular C—H⋯O hydrogen bonds.

## Related literature

For related literature, see: Blasko & Cordell (1988 ▶); Chen & Yu (2001 ▶); Liu *et al.* (2006 ▶); Parshikov *et al.* (2004 ▶, 2005 ▶, 2006 ▶); Zhan, Zhang *et al.* (2002 ▶); CDC (2007 ▶); Klayman (1985 ▶); TDR (2007 ▶); Zhan, Guo *et al.* (2002 ▶).
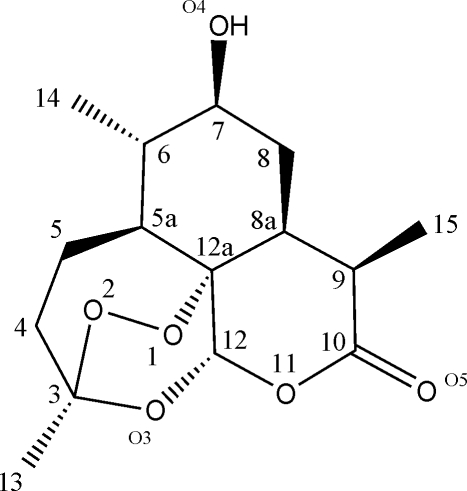


## Experimental

### 

#### Crystal data

C_15_H_22_O_6_
*M* *_r_* = 298.33
Orthorhombic, 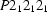

*a* = 6.3047 (2) Å
*b* = 9.1266 (2) Å
*c* = 24.5309 (6) Å
*V* = 1411.52 (6) Å^3^
*Z* = 4
Cu *K*α radiation
μ = 0.90 mm^−1^
*T* = 296 (2) K
0.23 × 0.15 × 0.12 mm

#### Data collection

Bruker SMART CCD diffractometer
Absorption correction: none
12572 measured reflections
2464 independent reflections
2456 reflections with *I* > 2σ(*I*)
*R*_int_ = 0.020

#### Refinement

*R*[*F* ^2^ > 2σ(*F* ^2^)] = 0.028
*wR*(*F* ^2^) = 0.072
*S* = 1.08
2464 reflections
194 parameters
H-atom parameters constrained
Δρ_max_ = 0.24 e Å^−3^
Δρ_min_ = −0.16 e Å^−3^
Absolute structure: Flack (1983 ▶), with 990 Friedel pairs
Flack parameter: 0.11 (14)

### 

Data collection: *SMART* (Bruker, 2002 ▶); cell refinement: *SAINT* (Bruker, 2002 ▶); data reduction: *SAINT*; program(s) used to solve structure: *SHELXS97* (Sheldrick, 2008 ▶); program(s) used to refine structure: *SHELXL97* (Sheldrick, 2008 ▶); molecular graphics: *SHELXTL* (Bruker, 2002 ▶); software used to prepare material for publication: *SHELXTL*.

## Supplementary Material

Crystal structure: contains datablocks I, global. DOI: 10.1107/S1600536808000251/hb2682sup1.cif

Structure factors: contains datablocks I. DOI: 10.1107/S1600536808000251/hb2682Isup2.hkl

Additional supplementary materials:  crystallographic information; 3D view; checkCIF report

## Figures and Tables

**Table 1:** Hydrogen-bond geometry (Å, °)

*D*—H⋯*A*	*D*—H	H⋯*A*	*D*⋯*A*	*D*—H⋯*A*
O4—H4⋯O4^i^	0.82	2.48	3.2488 (18)	156
C5*A*—H5*A*1⋯O3^ii^	0.98	2.53	3.4731 (16)	161
C5—H5*B*⋯O2^iii^	0.97	2.53	3.4571 (17)	159
C13—H13*B*⋯O2^iv^	0.96	2.44	3.3703 (18)	164

Symmetry codes: (i) 
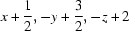
; (ii) 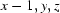; (iii) 
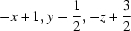
; (iv) 
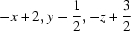
.

**Fig. 1.**
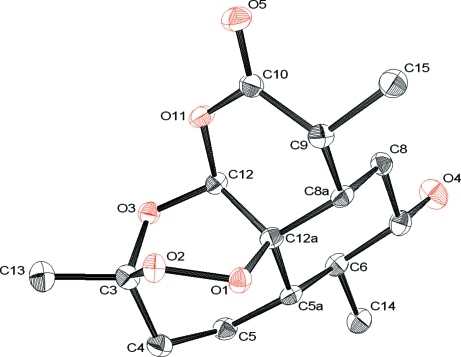
View of the molecular structure of (I) with displacement elipsoids drawn at the 50% probability level. Hydrogen atoms are omitted for clarity.

**Fig. 2.**
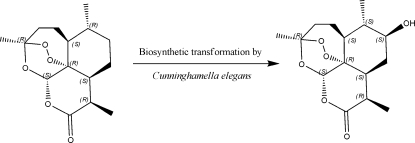
The formation of the title compound.

## supplementary crystallographic information

### Comment

The natural occurring sesquiterpene lactone endoperoxide artemisinin has been the subject of extensive research for its effective therapeutic action against multidrug-resistant *Plasmodium falciparum *strains (Klayman, 1985). Some of the reasons for this are the increasing number of people under risk of contracting malaria, the alarming spread of drug-resistant parasites (TDR, 2007) and the relatively complicated treatment protocols, with so many variables and no effective cure for the several strains of *Plasmodium* causing the disease (CDC, 2007).

One of the strategies used for increasing the bioavailability of artemisinin is its semi-synthetic transformation through microorganisms (Chen & Yu, 2001; Zhan, Guo *et al.*, 2002; Zhan, Zhang *et al.*, 2002; Liu *et al.*, 2006). The metabolites resulting from the action of several enzymes in selected strains of fungi can be further transformed in dimers or attached to other moieties for selective action and/or delivery.

Our group has been studying the microbial transformation of artemisinin for some years (Parshikov *et al.*, 2005; 2006) and we follow the numbering system of Blasko & Cordell (1988), and the CA Index Name. Some authors follow a different numbering system and call the title compound, (I), 9β-hydroxyartemisinin, rather than 7β-hydroxyartemisinin. Several well established methods of one-dimensional and two-dimensional NMR have already determined the configuration of artemisinin and most of its derivatives. The crystallographic data confirm the assignment of the chiral centers proposed in a previously published paper (Parshikov *et al.*, 2004). The configuration of the chiral centers in (I) are: C3 *R*, C5A S, C6 S, C7 S, C8A S, C9 *R*, C12 S, C12A *R*.

In the crystal of (I), a weak intermolecular O—H···O hydrogen bond links the molecules into chains and some short C—H···O contacts occur (Table 1).

### Experimental

7β-Hydroxyartemisinin was obtained following a method previously published by our group (Parshikov *et al.*, 2004). Well developed fungal mycelia of *Cunninghamella elegans* ATCC 9245 were removed from the surface of agar slants, suspended in 100 ml of sterilized water, and used to inoculate 1 liter of medium (400 g Sabouraud-dextrose, 300 g sucrose, 200 g peptone, in 20 liters of deionized water) in 4 liter shake flasks. The pH was adjusted to 6.5 using 0.1 N NaOH. Cultures were grown for 48 h on a rotary shaker at 301 K with shaking at 180 rpm. The resulting biomass was used as inocula for 1,000 ml of medium contained in 4 liter shake flasks that were again incubated for 48 h. 10 g of Artemisinin (Mediplantex, Vietnam) were dissolved in 400 ml of methanol (MeOH), filter-sterilized, and 20 ml were added to each flask to make the final concentration 500 mg/l. The cultures were returned to the shaker incubators (180 rpm) for an additional 14 days at 301 K. The cultures were harvested and the broth and mycelia were separated using coarse filter paper in a Büchner funnel. The mycelia were washed with water and discarded. The culture broth was extracted with three equal volumes of ethyl acetate (EtOAc) and evaporated under vacuum. The residues were dissolved in MeOH for analysis.

Thin layer chromatography (TLC) was performed on precoated silica gel G and GP Uniplates (Analtech, Newark, Del.) in EtOAc/hexanes (*v*/*v* 50:50). TLC plates were visualized with iodine, and *p*-anisaldehyde stain. Metabolites were purified by semi-preparative flash-chromatography carried out on silica gel 60 (SCI Adsorbents, Louisville, Ky.) using the EtOAc/hexanes solvent system in a gradient mode, eluting from 10–40% EtOAc, collecting 50 ml fractions with a flow rate of 30 ml/min.

Colourless needles of (I) were grown by slow evaporation of a solution in absolute ethanol.

### Refinement

The hydrogen atoms were placed in idealized locations (C—H = 0.96–0.98 Å, O—H = 0.82 Å) and refined as riding with *U*_iso_(H) = 1.2*U*_eq_(C) or 1.5*U*_eq_(methyl C, O).

### Crystal data

**Table:**

C_15_H_22_O_6_	*F*_000_ = 640
*M**_r_* = 298.33	*D*_x_ = 1.404 Mg m^−^^3^
Orthorhombic, *P*2_1_2_1_2_1_	Cu *K*α radiation λ = 1.54178 Å
Hall symbol: P 2ac 2ab	Cell parameters from 9907 reflections
*a* = 6.3047 (2) Å	θ = 3.6–66.0º
*b* = 9.1266 (2) Å	µ = 0.90 mm^−^^1^
*c* = 24.5309 (6) Å	*T* = 296 (2) K
*V* = 1411.52 (6) Å^3^	Needle, colourless
*Z* = 4	0.23 × 0.15 × 0.12 mm

### Data collection

**Table:**

Bruker SMART CCD diffractometer	2456 reflections with *I* > 2σ(*I*)
Monochromator: graphite	*R*_int_ = 0.020
*T* = 100 K	θ_max_ = 66.5º
ω scans	θ_min_ = 3.6º
Absorption correction: none	*h* = −7→7
12572 measured reflections	*k* = −10→10
2464 independent reflections	*l* = −28→29

### Refinement

**Table:**

Refinement on *F*^2^	Hydrogen site location: inferred from neighbouring sites
Least-squares matrix: full	H-atom parameters constrained
*R*[*F*^2^ > 2σ(*F*^2^)] = 0.028	*w* = 1/[σ^2^(*F*_o_^2^) + (0.0408*P*)^2^ + 0.3857*P*] where *P* = (*F*_o_^2^ + 2*F*_c_^2^)/3
*wR*(*F*^2^) = 0.072	(Δ/σ)_max_ = 0.001
*S* = 1.08	Δρ_max_ = 0.24 e Å^−^^3^
2464 reflections	Δρ_min_ = −0.16 e Å^−^^3^
194 parameters	Extinction correction: none
Primary atom site location: structure-invariant direct methods	Absolute structure: Flack (1983), 990 Friedel pairs
Secondary atom site location: difference Fourier map	Flack parameter: 0.11 (14)

### Special details

**Table:**

Geometry. All e.s.d.'s (except the e.s.d. in the dihedral angle between two l.s. planes)are estimated using the full covariance matrix. The cell e.s.d.'s are takeninto account individually in the estimation of e.s.d.'s in distances, anglesand torsion angles; correlations between e.s.d.'s in cell parameters are onlyused when they are defined by crystal symmetry. An approximate (isotropic)treatment of cell e.s.d.'s is used for estimating e.s.d.'s involving l.s. planes.
Refinement. Refinement of *F*^2^ against ALL reflections. The weighted *R*-factor *wR* andgoodness of fit *S* are based on *F*^2^, conventional *R*-factors *R* are basedon *F*, with *F* set to zero for negative *F*^2^. The threshold expression of*F*^2^ > σ(*F*^2^) is used only for calculating *R*-factors(gt) *etc*. and isnot relevant to the choice of reflections for refinement. *R*-factors basedon *F*^2^ are statistically about twice as large as those based on *F*, and *R*-factors based on ALL data will be even larger.

### Fractional atomic coordinates and isotropic or equivalent isotropic displacement parameters (Å^2^)

**Table:**

	*x*	*y*	*z*	*U*_iso_*/*U*_eq_	
O1	0.61177 (15)	1.07519 (10)	0.80390 (4)	0.0180 (2)	
O2	0.80303 (16)	1.07108 (10)	0.76947 (4)	0.0196 (2)	
O5	1.06564 (17)	1.28606 (12)	0.91615 (4)	0.0257 (2)	
O11	1.00751 (15)	1.07562 (11)	0.87615 (4)	0.0202 (2)	
O3	0.96938 (15)	0.88540 (11)	0.81679 (4)	0.0179 (2)	
O4	0.3981 (2)	0.79291 (13)	0.99868 (4)	0.0312 (3)	
H4	0.5183	0.7748	1.0095	0.047*	
C10	0.9351 (2)	1.20070 (15)	0.90000 (5)	0.0180 (3)	
C8A	0.5644 (2)	1.08876 (15)	0.89973 (5)	0.0164 (3)	
H8A	0.4185	1.1175	0.8910	0.020*	
C15	0.6384 (2)	1.33349 (16)	0.94762 (6)	0.0231 (3)	
H15A	0.6812	1.2917	0.9818	0.035*	
H15B	0.4877	1.3486	0.9477	0.035*	
H15C	0.7091	1.4256	0.9424	0.035*	
C6	0.4729 (2)	0.76793 (15)	0.90164 (6)	0.0196 (3)	
H6	0.6086	0.7227	0.9114	0.024*	
C7	0.4095 (2)	0.87131 (16)	0.94816 (6)	0.0211 (3)	
H7	0.2672	0.9086	0.9401	0.025*	
C3	0.8719 (2)	0.92375 (15)	0.76551 (5)	0.0189 (3)	
C13	1.0455 (3)	0.92467 (16)	0.72317 (6)	0.0244 (3)	
H13A	0.9850	0.9423	0.6879	0.037*	
H13B	1.1165	0.8316	0.7232	0.037*	
H13C	1.1456	1.0007	0.7315	0.037*	
C5	0.5912 (2)	0.74642 (15)	0.80360 (6)	0.0205 (3)	
H5A	0.6983	0.6846	0.8203	0.025*	
H5B	0.4763	0.6831	0.7919	0.025*	
C9	0.6978 (2)	1.22918 (15)	0.90130 (6)	0.0177 (3)	
H9	0.6651	1.2817	0.8675	0.021*	
C14	0.3095 (2)	0.64461 (16)	0.89482 (6)	0.0241 (3)	
H14A	0.2810	0.6009	0.9296	0.036*	
H14B	0.3647	0.5716	0.8705	0.036*	
H14C	0.1806	0.6842	0.8801	0.036*	
C4	0.6880 (2)	0.81822 (16)	0.75307 (6)	0.0207 (3)	
H4A	0.5777	0.8717	0.7340	0.025*	
H4B	0.7389	0.7419	0.7289	0.025*	
C12A	0.6419 (2)	0.98999 (15)	0.85346 (6)	0.0164 (3)	
C12	0.8748 (2)	0.95112 (15)	0.86157 (6)	0.0166 (3)	
H12	0.8821	0.8810	0.8918	0.020*	
C8	0.5571 (2)	1.00298 (15)	0.95338 (5)	0.0185 (3)	
H8B	0.6987	0.9698	0.9627	0.022*	
H8C	0.5074	1.0665	0.9824	0.022*	
C5A	0.5049 (2)	0.85116 (16)	0.84744 (5)	0.0171 (3)	
H5A1	0.3644	0.8832	0.8352	0.021*	

### Atomic displacement parameters (Å^2^)

**Table:**

	*U*^11^	*U*^22^	*U*^33^	*U*^12^	*U*^13^	*U*^23^
O1	0.0161 (5)	0.0194 (5)	0.0185 (5)	0.0033 (4)	0.0003 (4)	0.0022 (4)
O2	0.0198 (5)	0.0179 (5)	0.0212 (5)	0.0016 (4)	0.0043 (4)	0.0019 (4)
O5	0.0209 (5)	0.0243 (5)	0.0318 (5)	−0.0053 (5)	−0.0020 (4)	−0.0077 (4)
O11	0.0135 (5)	0.0206 (5)	0.0266 (5)	−0.0006 (4)	−0.0026 (4)	−0.0048 (4)
O3	0.0156 (4)	0.0185 (5)	0.0196 (5)	0.0031 (4)	−0.0004 (4)	−0.0017 (4)
O4	0.0419 (7)	0.0276 (6)	0.0240 (5)	−0.0063 (5)	0.0054 (5)	0.0044 (5)
C10	0.0188 (7)	0.0186 (7)	0.0166 (6)	−0.0016 (6)	−0.0004 (5)	−0.0003 (5)
C8A	0.0130 (6)	0.0155 (6)	0.0208 (6)	0.0007 (6)	−0.0018 (5)	−0.0004 (5)
C15	0.0225 (7)	0.0183 (7)	0.0283 (7)	−0.0007 (6)	0.0008 (6)	−0.0034 (6)
C6	0.0174 (7)	0.0171 (7)	0.0244 (7)	−0.0005 (6)	−0.0004 (6)	0.0021 (6)
C7	0.0194 (7)	0.0214 (7)	0.0225 (7)	−0.0011 (6)	0.0033 (6)	0.0025 (6)
C3	0.0213 (7)	0.0165 (7)	0.0189 (6)	0.0020 (6)	−0.0004 (6)	−0.0002 (5)
C13	0.0279 (8)	0.0201 (7)	0.0253 (7)	0.0001 (6)	0.0054 (6)	−0.0001 (6)
C5	0.0205 (7)	0.0174 (6)	0.0234 (7)	−0.0033 (6)	−0.0011 (6)	−0.0029 (6)
C9	0.0178 (7)	0.0160 (7)	0.0192 (6)	0.0006 (6)	−0.0018 (5)	0.0000 (5)
C14	0.0235 (7)	0.0193 (7)	0.0295 (8)	−0.0029 (7)	0.0009 (6)	0.0020 (6)
C4	0.0226 (7)	0.0200 (7)	0.0196 (6)	0.0004 (6)	−0.0024 (6)	−0.0023 (5)
C12A	0.0157 (7)	0.0159 (6)	0.0175 (6)	0.0009 (6)	−0.0024 (5)	0.0018 (5)
C12	0.0146 (6)	0.0154 (6)	0.0198 (7)	−0.0007 (5)	−0.0002 (5)	0.0003 (5)
C8	0.0189 (7)	0.0184 (7)	0.0181 (6)	0.0012 (6)	0.0009 (6)	−0.0014 (5)
C5A	0.0126 (6)	0.0186 (7)	0.0202 (6)	−0.0006 (5)	−0.0027 (5)	−0.0004 (5)

### Geometric parameters (Å, °)

**Table:**

O1—C12A	1.4556 (16)	C7—C8	1.525 (2)
O1—O2	1.4727 (13)	C7—H7	0.9800
O2—C3	1.4164 (17)	C3—C13	1.509 (2)
O5—C10	1.2003 (18)	C3—C4	1.538 (2)
O11—C10	1.3615 (17)	C13—H13A	0.9600
O11—C12	1.4558 (17)	C13—H13B	0.9600
O3—C12	1.3866 (17)	C13—H13C	0.9600
O3—C3	1.4429 (16)	C5—C4	1.529 (2)
O4—C7	1.4328 (17)	C5—C5A	1.5383 (19)
O4—H4	0.8200	C5—H5A	0.9700
C10—C9	1.5193 (19)	C5—H5B	0.9700
C8A—C12A	1.5297 (18)	C9—H9	0.9800
C8A—C8	1.5320 (18)	C14—H14A	0.9600
C8A—C9	1.5333 (19)	C14—H14B	0.9600
C8A—H8A	0.9800	C14—H14C	0.9600
C15—C9	1.5289 (19)	C4—H4A	0.9700
C15—H15A	0.9600	C4—H4B	0.9700
C15—H15B	0.9600	C12A—C12	1.5234 (19)
C15—H15C	0.9600	C12A—C5A	1.5405 (19)
C6—C7	1.5336 (19)	C12—H12	0.9800
C6—C14	1.535 (2)	C8—H8B	0.9700
C6—C5A	1.5444 (18)	C8—H8C	0.9700
C6—H6	0.9800	C5A—H5A1	0.9800
			
C12A—O1—O2	111.00 (9)	C5A—C5—H5A	108.2
C3—O2—O1	108.33 (9)	C4—C5—H5B	108.2
C10—O11—C12	124.56 (11)	C5A—C5—H5B	108.2
C12—O3—C3	113.74 (10)	H5A—C5—H5B	107.4
C7—O4—H4	109.5	C10—C9—C15	111.29 (12)
O5—C10—O11	117.15 (13)	C10—C9—C8A	113.38 (11)
O5—C10—C9	123.87 (13)	C15—C9—C8A	113.89 (11)
O11—C10—C9	118.85 (12)	C10—C9—H9	105.8
C12A—C8A—C8	110.23 (11)	C15—C9—H9	105.8
C12A—C8A—C9	109.62 (11)	C8A—C9—H9	105.8
C8—C8A—C9	114.95 (11)	C6—C14—H14A	109.5
C12A—C8A—H8A	107.2	C6—C14—H14B	109.5
C8—C8A—H8A	107.2	H14A—C14—H14B	109.5
C9—C8A—H8A	107.2	C6—C14—H14C	109.5
C9—C15—H15A	109.5	H14A—C14—H14C	109.5
C9—C15—H15B	109.5	H14B—C14—H14C	109.5
H15A—C15—H15B	109.5	C5—C4—C3	114.09 (11)
C9—C15—H15C	109.5	C5—C4—H4A	108.7
H15A—C15—H15C	109.5	C3—C4—H4A	108.7
H15B—C15—H15C	109.5	C5—C4—H4B	108.7
C7—C6—C14	110.94 (12)	C3—C4—H4B	108.7
C7—C6—C5A	111.84 (11)	H4A—C4—H4B	107.6
C14—C6—C5A	110.77 (11)	O1—C12A—C12	111.07 (11)
C7—C6—H6	107.7	O1—C12A—C8A	105.26 (10)
C14—C6—H6	107.7	C12—C12A—C8A	110.38 (11)
C5A—C6—H6	107.7	O1—C12A—C5A	106.63 (10)
O4—C7—C8	110.58 (12)	C12—C12A—C5A	111.18 (11)
O4—C7—C6	110.46 (12)	C8A—C12A—C5A	112.12 (11)
C8—C7—C6	112.84 (12)	O3—C12—O11	106.56 (11)
O4—C7—H7	107.6	O3—C12—C12A	114.31 (11)
C8—C7—H7	107.6	O11—C12—C12A	113.85 (11)
C6—C7—H7	107.6	O3—C12—H12	107.2
O2—C3—O3	107.52 (10)	O11—C12—H12	107.2
O2—C3—C13	105.32 (11)	C12A—C12—H12	107.2
O3—C3—C13	107.01 (11)	C7—C8—C8A	110.41 (11)
O2—C3—C4	112.14 (12)	C7—C8—H8B	109.6
O3—C3—C4	110.01 (11)	C8A—C8—H8B	109.6
C13—C3—C4	114.44 (12)	C7—C8—H8C	109.6
C3—C13—H13A	109.5	C8A—C8—H8C	109.6
C3—C13—H13B	109.5	H8B—C8—H8C	108.1
H13A—C13—H13B	109.5	C5—C5A—C12A	112.32 (11)
C3—C13—H13C	109.5	C5—C5A—C6	110.02 (11)
H13A—C13—H13C	109.5	C12A—C5A—C6	113.30 (11)
H13B—C13—H13C	109.5	C5—C5A—H5A1	106.9
C4—C5—C5A	116.20 (12)	C12A—C5A—H5A1	106.9
C4—C5—H5A	108.2	C6—C5A—H5A1	106.9

### Hydrogen-bond geometry (Å, °)

**Table:**

*D*—H···*A*	*D*—H	H···*A*	*D*···*A*	*D*—H···*A*
O4—H4···O4^i^	0.82	2.48	3.2488 (18)	156
C5A—H5A1···O3^ii^	0.98	2.53	3.4731 (16)	161
C5—H5B···O2^iii^	0.97	2.53	3.4571 (17)	159
C13—H13B···O2^iv^	0.96	2.44	3.3703 (18)	164

Symmetry codes: (i) *x*+1/2, −*y*+3/2, −*z*+2; (ii) *x*−1, *y*, *z*; (iii) −*x*+1, *y*−1/2, −*z*+3/2; (iv) −*x*+2, *y*−1/2, −*z*+3/2.
